# 48. Time Between Viral Loads for Suppressed and Non-Suppressed People with HIV During the COVID-19 Pandemic Compared to Pre-Pandemic

**DOI:** 10.1093/ofid/ofab466.048

**Published:** 2021-12-04

**Authors:** Walid El-Nahal, Nicola Shen, Catherine Lesko, Anthony Fojo, Bryan Lau, Jeanne Keruly, Yukari C Manabe, Joyce Jones, Richard Moore, Kelly Gebo, Geetanjali Chander

**Affiliations:** 1 Johns Hopkins University School of Medicine, Baltimore, Maryland; 2 Johns Hopkins Bloomberg School of Public Health, Baltimore, Maryland; 3 The Johns Hopkins University School of Medicine, Baltimore, MD; 4 Johns Hopkins, Baltimore, MD; 5 Johns Hopkins University, Baltimore, MD

## Abstract

**Background:**

During the COVID-19 pandemic, patients at the John G. Bartlett Specialty practice experienced disruptions in viral load (VL) monitoring due to 1) conversion to telemedicine visits and 2) closure of the onsite lab from March 16-July 13, 2021. We described the impact of the pandemic on VL monitoring.

**Methods:**

We measured time from all index VLs collected during 3 periods: January 1, 2019 to March 15, 2020 (pre-pandemic); March 16 to July 12, 2020 (pandemic, closed onsite lab); and July 13 to December 31, 2020 (pandemic, open onsite lab) until a subsequent VL, 1 year after the index VL, or administrative censoring on December 31, 2020, whichever came first. We classified follow-up time according to these periods (treating period as a time-varying variable). We report hazard ratios (HRs) and 95% Confidence Intervals (CI) from a Cox proportional hazards model comparing the hazard of a VL during the pandemic periods to the pre-pandemic period, stratified by whether the index VL was suppressed (≤200 copies/mL). We tested for interactions between patient characteristics (age, sex at birth, race, ethnicity, and recent substance use) and period, to investigate differential effects of the pandemic on delayed VL.

**Results:**

After 7,760 suppressed VL measurements, median times to subsequent VL during the pre-pandemic, pandemic (closed lab) and pandemic (open lab) periods, were 4.6 (HR=1.0), 8.9 (HR=0.34, CI:0.30, 0.37), and 5.8 (HR=0.73, CI:0.68,0.78) months respectively. After 1,025 non-suppressed VL measurements, median times to subsequent VL were 2.0 (HR=1.0), 3.9 (HR=0.57, CI:0.42,0.79), and 2.1 (HR=0.92, CI:0.76,1.10) months respectively. Time to subsequent VL after an index suppressed VL was less affected by the pandemic for patients who are white; had private insurance; or had no recent cocaine or heroin use. The effect of the pandemic on time to subsequent VL after a non-suppressed index VL did not significantly differ across patient characteristics.

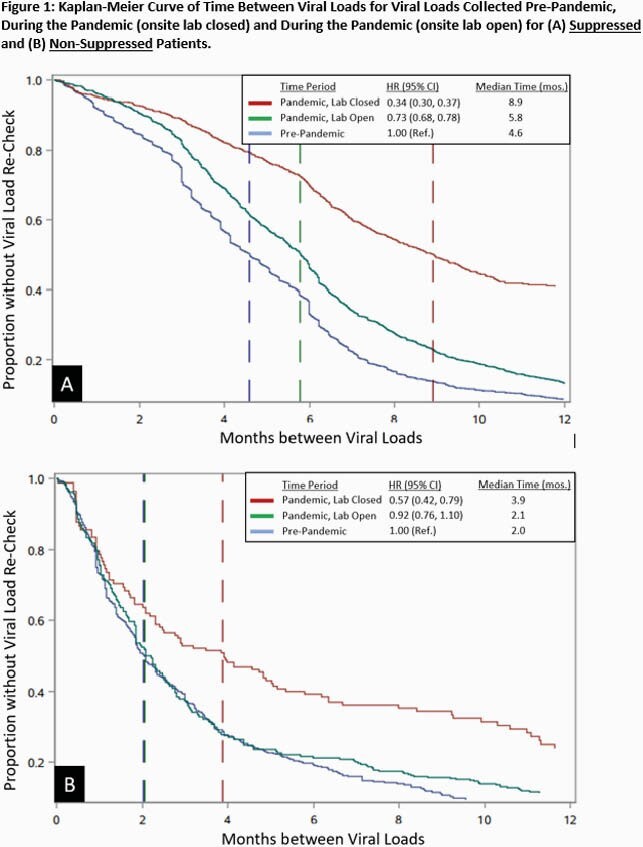

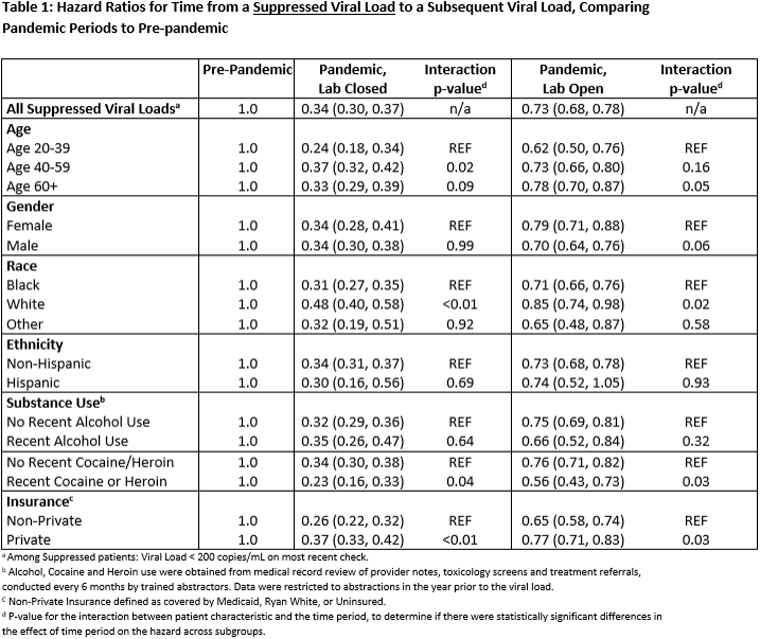

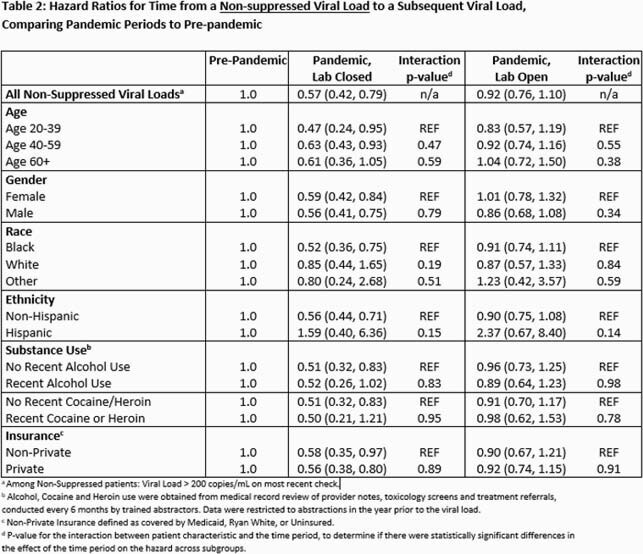

**Conclusion:**

Onsite lab closure disrupted VL collection for all groups. Once the onsite lab opened, the pandemic period was still associated with a delay among suppressed patients, but not non-suppressed patients. Further studies are needed to investigate if these delays are associated with lapses in viral suppression.

**Disclosures:**

**All Authors**: No reported disclosures

